# Low T3 Syndrome in Acute Myocardial Infarction: Association With Cardiac Biomarkers and Implications for Myocardial Injury Severity

**DOI:** 10.7759/cureus.113220

**Published:** 2026-07-23

**Authors:** Piyush K Singh, Supriya, Rekha Choudhary, Udayakumar Rangasamy, Carlos Noel C Menezes

**Affiliations:** 1 Biochemistry, Mahatma Vidur Autonomous State Medical College, Bijnor, IND; 2 Community Medicine, Uttar Pradesh University of Medical Sciences, Saifai, IND; 3 Biochemistry, Goa Medical College, Bambolim, IND

**Keywords:** acute myocardial infarction, cardiac biomarkers, ck-mb, low t3 syndrome, thyroid-stimulating hormone

## Abstract

Background

An acute myocardial infarction (AMI) triggers widespread inflammatory, neuroendocrine, and metabolic shifts across the body. Deviations in thyroid hormone regulation, particularly the development of low triiodothyronine (T3) syndrome, are frequently observed during acute cardiovascular events and may mirror the intensity of the underlying physiological stress.

Objective

The objective of the study is to evaluate the prevalence of low T3 syndrome in individuals presenting with an initial episode of AMI and to investigate its independent relationship with biochemical indicators of myocardial damage.

Methods

This prospective observational investigation featured 100 consecutive patients aged 30 to 70 years who were admitted with their first AMI at a tertiary care teaching institution. Cases of AMI were confirmed using clinical presentations, electrocardiographic variations, and elevated troponin I and/or creatine kinase-MB (CK-MB) concentrations. Circulating blood samples collected within roughly six hours of symptom onset were evaluated for free T3 (fT3), free thyroxine (fT4), thyroid-stimulating hormone (TSH), troponin I, and CK-MB using a chemiluminescent microparticle immunoassay. Low T3 syndrome was identified by diminished serum fT3 concentrations alongside relatively steady fT4 and TSH profiles. Skewed biomarker distributions (fT3 and TSH) were reported as median (interquartile range) and subjected to a logarithmic transformation (log10) prior to multivariable analysis. Statistical relationships were evaluated via Pearson correlation analysis and a multivariable linear regression model using log-transformed troponin I as the primary dependent variable.

Results

The cohort presented a mean age of 56.3 ± 14.3 years, with male patients representing 65% of the total group. Low T3 syndrome was documented in 30% of the study population. In the multivariable linear regression model adjusting for baseline demographic confounders (age and sex), both log-transformed TSH (β = 0.33, p = 0.001) and log-transformed fT3 (β = 0.28, p = 0.003) emerged as significant independent predictors of myocardial injury severity (log-troponin I).

Conclusion

Low T3 syndrome occurred frequently in patients experiencing a first-episode AMI. Baseline thyroid-axis alterations, specifically log-TSH and log-fT3, are significantly and independently associated with biochemical markers of myocardial injury at admission. However, as these early neuroendocrine shifts represent a cross-sectional baseline snapshot, their direct relationship with structural infarct size, ventricular dysfunction, or long-term clinical prognosis requires further validation in larger trials.

## Introduction

Even with ongoing advances in reperfusion methods, clinical pharmacotherapy, and specialized coronary care facilities, acute myocardial infarction (AMI) remains a major contributor to global morbidity and mortality [1,2]. This burden is particularly striking in India and other South Asian populations, where coronary artery disease often presents at a younger age, leading to substantial premature mortality and prolonged disability. Data from Indian clinical registries continue to highlight challenges related to delayed presentation, variations in treatment strategies, and disparities in outcomes, underscoring the need for improved risk stratification and a better understanding of the biological changes that occur during acute ischemic events [3,4].

While the underlying trigger of an AMI is acute coronary plaque disruption coupled with thrombotic occlusion, its clinical trajectory is dictated by a wider systemic response. This response encompasses inflammation, sympathetic nervous system activation, oxidative stress, hemodynamic instability, and metabolic adjustments [1,5]. These systemic dynamics heavily influence how an infarct evolves, potentially altering the degree of myocardial damage, ventricular impairment, and eventual recovery. Standard cardiac indicators like troponin and creatine kinase-MB (CK-MB) remain fundamental for clinical diagnosis and injury evaluation; however, they fail to reflect the broader neuroendocrine and metabolic reactions that occur alongside an AMI [1].

Thyroid hormones exert an essential influence on cardiovascular biology. As the principal biologically active thyroid hormone, triiodothyronine (T3) regulates myocardial contractility, diastolic relaxation, heart rate, vascular resistance, endothelial cell function, and intracellular calcium utilization through both genomic and non-genomic pathways [5,6]. Consequently, minor deviations in available thyroid hormones can disrupt cardiovascular equilibrium. Both clinical and subclinical thyroid anomalies have been linked to modifications in cardiovascular structure and performance, and a growing body of evidence indicates that thyroid hormone signaling is intricately tied to cardiovascular adaptation under states of severe stress [5-7].

During acute medical crises, thyroid hormone regulation often transitions into a distinctive state known as low T3 syndrome or non-thyroidal illness syndrome. This condition is classically defined by lower levels of circulating T3 with relatively preserved thyroxine (T4) and thyroid-stimulating hormone (TSH) parameters, alongside elevated reverse T3 levels when assessed [7-9]. The causative mechanism is multifactorial, involving restricted peripheral conversion of T4 to T3 driven by modified deiodinase activity, cytokine-driven disruptions in thyroid hormone pathways, dysregulation of the hypothalamic-pituitary-thyroid axis, and variations in hormone-binding dynamics [8,9]. While these alterations may represent a protective, energy-conserving adaptation to acute physiological distress, maladaptive tissue-level hypothyroidism has also been observed in specific scenarios [8-10].

This endocrine response holds notable clinical relevance in the setting of AMI due to the profound neuroendocrine surge that accompanies an AMI. Marked spikes in catecholamines, activation of the renin-angiotensin-aldosterone system, the systemic release of inflammatory cytokines, and localized tissue hypoxia can each individually interfere with thyroid hormone pathways and hypothalamic-pituitary-thyroid signaling [5,7,8]. From a mechanistic standpoint, these interrelated pathways clarify why low T3 states occur during an AMI and help explain established connections with compromised ventricular mechanics and poor clinical outcomes [6,11].

A variety of clinical investigations have analyzed thyroid axis variations during acute coronary syndromes. Initial work by Iervasi et al. revealed that low T3 syndrome serves as a powerful prognostic indicator in individuals with cardiac disease, a finding that subsequent studies validated within acute coronary populations [11]. Su et al. observed that the presence of low T3 syndrome refined risk prediction models for in-hospital cardiovascular mortality during an AMI and correlated with more pronounced myocardial damage [12]. Similarly, Yazıcı et al. determined that low T3 levels were tied to one-year mortality rates in cases of non-ST-elevation acute coronary syndromes [13,14]. Furthermore, Jabbar et al. noted that thyroid irregularities are relatively frequent among individuals presenting with an AMI and carry notable prognostic value [15]. A sentiment echoed by Abdulaziz Qari regarding thyroid hormone shifts in broader acute coronary syndrome cohorts [16].

Despite this expanding focus on thyroid-cardiovascular connections, notable insights are still missing. Much of the published literature centers on endpoints like mortality, heart failure development, or collective cardiovascular events, rather than investigating the direct link between thyroid function parameters and biochemical indicators of myocardial damage at the exact time of AMI presentation. Investigations evaluating free T3 (fT3), free T4 (fT4), and TSH against troponin I and CK-MB remain relatively sparse, particularly among Indian cohorts experiencing an initial AMI episode [12,13]. Uncovering these connections could offer deeper insights into the neuroendocrine response to acute ischemic injury and clarify whether assessing thyroid function yields valuable supplementary biological data during early AMI triage [13,15-18]. Consequently, this study was organized to establish the prevalence of low T3 syndrome in patients presenting with an initial episode of AMI and to analyze its relationship with standard cardiac biomarkers of myocardial damage.

## Materials and methods

Study design and setting

This hospital-based, prospective observational study was conducted over a 12-month period from January 2021 to December 2021. The clinical work was carried out in the emergency medicine units and intensive care facilities of Goa Medical College and Hospital, a tertiary-level teaching institution in Goa, India. The study was performed through the collaborative efforts of the Departments of Biochemistry, Medicine, and Cardiology. This was a non-funded study.

Study population and sample size

The study population comprised 100 consecutive patients aged 30 to 70 years who presented during the study period with their first episode of AMI and fulfilled the predefined eligibility criteria. The sample size was determined using a time-bound consecutive sampling approach rather than a prior sample size calculation. All eligible patients who presented within the fixed study duration and met the inclusion and exclusion criteria were enrolled. Although no formal a priori sample size estimation was performed, a post hoc power analysis was conducted to assess adequacy of the final cohort. At a two-tailed significance level of 0.05, the sample size of 100 patients provided a statistical power of 86.1% to detect the moderate positive correlation observed between serum fT3 and troponin I, and a power of 99.9% for the stronger positive correlation between TSH and CK-MB. The cohort size was also considered adequate for multivariable linear regression analysis with four independent predictors and an overall significant model fit [1].

Inclusion and exclusion criteria

Patients were included if they were between 30 and 70 years of age and were admitted with a first episode of AMI confirmed by clinical presentation, electrocardiographic changes, and elevated troponin I and/or CK-MB levels in accordance with the Fourth Universal Definition of Myocardial Infarction. Patients were excluded if they had a known history of thyroid disorder, were receiving thyroid-modifying drugs or systemic corticosteroids, had recent exposure to iodinated contrast media, or were pregnant or lactating. Patients with renal insufficiency were excluded if serum creatinine exceeded 1.5 mg/dL. Patients with hepatic insufficiency were excluded if serum transaminases, including aspartate aminotransferase (AST) or alanine aminotransferase (ALT), were elevated to more than three times the upper reference limit, or if total bilirubin was greater than 2 mg/dL. These biochemical thresholds were used to define renal and hepatic dysfunction objectively for the purpose of patient selection.

Data collection procedures

Initial demographic characteristics and clinical metrics were systematically gathered at the time of admission using a structured case recording form. Peripheral venous blood samples were drawn prior to initiating definitive therapeutic interventions, with a median time of 3.5 hours (interquartile range (IQR): 2.0-5.0 hours) from the reported onset of chest discomfort.

Laboratory investigations

Serum concentrations of fT3, fT4, TSH, troponin I, and CK-MB were measured using chemiluminescent microparticle immunoassay (CMIA) techniques on an Abbott ARCHITECT analyzer platform (Abbott Laboratories, IL, USA). All biochemical assessments were executed within the institution’s central clinical biochemistry facility according to the vendor's protocol, utilizing standardized internal quality control benchmarks.

Definitions of low T3 syndrome

Patients were categorized based on specific serum reference ranges established by our laboratory. Low T3 syndrome (also referred to as non-thyroidal illness syndrome) was explicitly defined as a subnormal serum fT3 level (<2.0 pg/mL) accompanied by normal to low levels of fT4 and a non-elevated TSH concentration, in the absence of preexisting intrinsic pituitary or thyroid gland disease. Patients falling entirely within normal reference intervals for all three parameters were classified as euthyroid [9,10].

Statistical analysis

Collected data were entered into Microsoft Excel (Microsoft Corp., Redmond, WA, USA) and analyzed using SPSS version 22.0 (IBM Corp., Armonk, NY, USA). Continuous variables were assessed for distributional normality using the Shapiro-Wilk test. Normally distributed variables are presented as mean ± standard deviation, whereas non-normally distributed variables are presented as median (IQR). Because serum fT3, TSH, troponin I, and CK-MB demonstrated a non-normal distribution, bivariate associations were evaluated using Spearman's rank correlation coefficient (ρ). For multivariable linear regression analysis, skewed continuous variables were logarithmically transformed (log10) to approximate normality and satisfy the assumptions of linear regression. A multivariable linear regression model was subsequently performed to examine the independent association of log-transformed thyroid parameters with log-transformed troponin I as the primary dependent variable, after adjustment for age, sex, and fT4. A two-tailed p-value < 0.05 was considered statistically significant.

Ethical considerations

The research was executed in strict adherence to the ethical principles of the Declaration of Helsinki. Formal approval from the Institutional Ethics Committee was secured at Goa Medical College before starting the study, and signed informed consent was obtained from every patient prior to baseline enrollment. The authors received no external funding and have no competing financial interests to disclose.

## Results

The final data analysis included 100 patients with a verified diagnosis of AMI. The baseline mean age of the group was 56.3 ± 14.3 years, and men made up 65.0% of the cohort. These baseline demographic findings are outlined in Table 1.

**Table 1:** Baseline characteristics of the study population SD: standard deviation

Variable	Value
Total patients, n	100
Age, years (mean ± SD)	56.3 ± 14.3
Male, n (%)	65 (65.0)
Female, n (%)	35 (35.0)

The mean fT4 level was 1.21 ± 0.74 ng/dL, while the median (IQR) values for fT3 and TSH were 2.20 (1.75-2.90) pg/mL and 4.20 (3.10-6.10) µIU/mL, respectively. Low T3 syndrome was identified in 30 patients (30%), whereas 70 patients (70%) were euthyroid (Table 2).

**Table 2:** Thyroid function profile and prevalence of low T3 syndrome SD: standard deviation; fT3: free triiodothyronine; fT4: free thyroxine; TSH: thyroid-stimulating hormone; IQR: interquartile range

Parameter	Value
fT3, pg/mL (median (IQR))	2.20 (1.75-2.90)
fT4, ng/dL (mean ± SD)	1.21 ± 0.74
TSH, μIU/mL median (IQR)	4.20 (3.10-6.10)
Low T3 syndrome, n (%)	30 (30%)
Euthyroid, n (%)	70 (70%)

**Figure 1:**
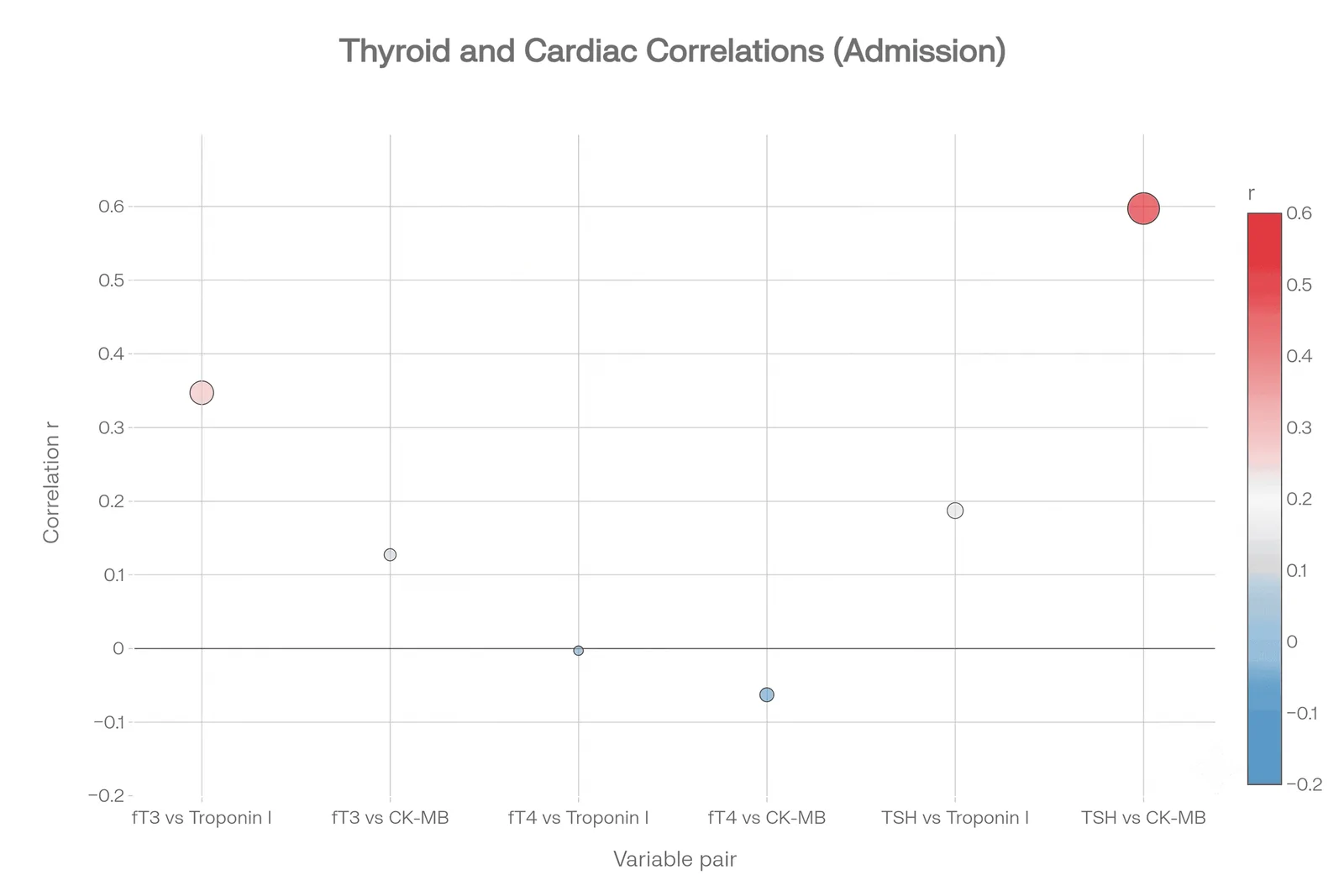
Correlation of thyroid function parameters with cardiac biomarkers at admission. Bubble size and color represent the strength and direction of Spearman's rank correlation coefficients (ρ) fT3: free triiodothyronine; fT4: free thyroxine; TSH: thyroid-stimulating hormone; CK-MB: creatine kinase-MB

Spearman's rank correlation coefficients (ρ) were used to assess linear associations between continuous variables. A p-value < 0.05 was considered statistically significant (Table 3, Figure 1).

**Table 3:** Correlation between thyroid function parameters and cardiac biomarkers in patients with acute myocardial infarction fT3: free triiodothyronine; fT4: free thyroxine; TSH: thyroid-stimulating hormone; CK-MB: creatine kinase-MB

Thyroid parameter	Cardiac biomarker	Spearman's rank correlation coefficients (ρ)	p-value
fT3	Troponin I	0.30	0.003
fT3	CK-MB	0.08	0.430
fT4	Troponin I	-0.05	0.620
fT4	CK-MB	-0.11	0.275
TSH	Troponin I	0.14	0.165
TSH	CK-MB	0.55	<0.001

To evaluate the independent predictive capacity of baseline thyroid parameters on myocardial injury severity, a full multivariable linear regression model was constructed using log-transformed troponin I as the dependent variable (Table 4). After adjusting for age and sex, the full model accounted for 38.5% of the variance in biomarker levels (R^2^ = 0.385, p < 0.001; R^2^ = 0.385, p < 0.001; R^2^ = 0.385, p < 0.001). Both log-transformed fT3 (β = 0.28, t = 3.09, p = 0.003; β = 0.28, t = 3.09, p = 0.003; β = 0.28, t = 3.09, p = 0.003) and log-transformed TSH (β = 0.33, t = 3.43, p = 0.001; β = 0.33, t = 3.43, p = 0.001; β = 0.33, t = 3.43, p = 0.001) remained significant independent predictors within the full model, whereas age, sex, and fT4 levels did not exhibit statistically significant associations (p > 0.05; p > 0.05; p > 0.05).

**Table 4:** Multivariable linear regression model predicting myocardial injury (log-troponin I) In the multivariable linear regression analysis adjusted for age, sex, and fT4 (R² = 0.385; adjusted R² = 0.352; F = 11.78; p < 0.001), log-transformed TSH (β = 0.33, B = 0.48, p = 0.001) and log-transformed fT3 (β = 0.28, B = 0.34, p = 0.003) remained independently associated with log-transformed troponin I levels at presentation. fT3: free triiodothyronine; fT4: free thyroxine; TSH: thyroid-stimulating hormone

Variable	Unstandardized coefficient (B)	Standard error (SE)	Standardized coefficient (β)	t-statistic	p-value	95% confidence interval (CI)
(Constant)	1.84	0.42	-	4.38	<0.001	(1.01 to 2.67)
Age	0.01	0.01	0.08	0.82	0.414	(-0.01 to 0.03)
Sex (male)	0.12	0.09	0.11	1.33	0.187	(-0.06 to 0.30)
Log-fT3	0.34	0.11	0.28	3.09	0.003	(0.12 to 0.56)
fT4	-0.05	0.07	-0.06	-0.71	0.479	(-0.19 to 0.09)
Log-TSH	0.48	0.14	0.33	3.43	0.001	(0.20 to 0.76)

## Discussion

In this prospective observational study, low T3 syndrome was identified in 30% of patients presenting with a first episode of AMI. The principal finding was that, in a multivariable linear regression analysis adjusted for age, sex, and fT4 (R² = 0.385; adjusted R² = 0.352; F = 11.78; p < 0.001), both log-transformed TSH (β = 0.33, B = 0.48, p = 0.001) and log-transformed fT3 (β = 0.28, B = 0.34, p = 0.003) remained independently associated with log-transformed troponin I levels at presentation. These findings indicate that baseline thyroid-axis alterations are independently associated with the biochemical severity of myocardial injury at admission, even after adjustment for demographic variables and circulating fT4 levels. However, because these findings are derived from a single-time-point biochemical assessment obtained at presentation, they suggest that thyroid-axis alterations accompany AMI but do not establish a direct relationship with structural infarct size, ventricular dysfunction, or long-term clinical prognosis.

The observed prevalence of low T3 syndrome in this cohort is broadly consistent with previous reports showing that thyroid-axis abnormalities are common during acute coronary syndromes and critical illness states. Prior studies by Su et al., Jabbar et al., and Abdulaziz Qari have similarly documented frequent thyroid hormone disturbances among patients with AMI or acute coronary syndromes, although the exact prevalence has varied according to study population, assay method, diagnostic thresholds, and timing of blood sampling. The 30% prevalence noted in the present study may therefore reflect the combined influence of early sampling after symptom onset, inclusion of only first-episode AMI cases, and local patient characteristics.

The biological basis for low T3 syndrome during AMI is likely multifactorial. Acute ischemic injury is accompanied by a surge in catecholamines, inflammatory cytokines, oxidative stress, tissue hypoxia, and neurohormonal activation, all of which may alter deiodinase activity, disrupt hypothalamic-pituitary-thyroid regulation, and impair peripheral conversion of T4 to T3 [7-10]. These mechanisms support the concept that thyroid hormone disturbances in AMI are part of a broader systemic adaptive response to critical illness rather than an isolated endocrine abnormality [7-10].

A major finding of the present study was the independent positive association of both log-TSH and log-fT3 with log-troponin I. This observation suggests that early changes within the hypothalamic-pituitary-thyroid axis closely parallel the biochemical intensity of myocardial injury during the early phase of AMI. However, this interpretation must remain conservative. Cardiac biomarkers were used here as biochemical surrogates of myocardial injury, and the study did not include direct measures of infarct size, such as cardiac magnetic resonance imaging, detailed echocardiographic parameters, or left ventricular ejection fraction, nor did it assess inflammatory markers or longitudinal clinical outcomes. Accordingly, these independent associations should be viewed as cross-sectional biochemical correlations rather than proof of structural myocardial damage or prognostic significance.

The positive independent association between log-fT3 and log-troponin I requires careful context in light of prior literature, in which low T3 states are generally associated with greater illness severity and poorer cardiovascular outcomes. Several explanations may account for this early presentation pattern. First, thyroid hormone and biomarker responses during AMI are highly dynamic, and a single baseline sample obtained early after admission may not capture later, progressive declines in fT3 or the full temporal evolution of troponin release. Second, troponin values in AMI can be strongly influenced by the exact time from symptom onset to sampling, baseline patient characteristics, and early assay kinetics, all of which alter the pattern observed at presentation. For these reasons, this independent biochemical association at admission should be regarded as an acute cross-sectional correlate rather than a tracking of longitudinal disease evolution.

This cautious interpretation is further supported by the analytic structure of the study. The investigation was designed as a cross-sectional admission-based assessment and not as a longitudinal or prospective outcome-oriented follow-up trial. As a result, it was not possible to evaluate serial changes in thyroid hormones or cardiac biomarkers over time, nor to determine how these relationships shift after stabilization, reperfusion, or clinical recovery. Furthermore, because relevant acute clinical confounders-including the precise distribution of ST-segment elevation myocardial infarction (STEMI) versus non-STEMI (NSTEMI) presentation, Killip class stratification, emergency reperfusion strategies, and acute pharmacotherapy exposure-could not be uniformly tracked across the database, residual confounding cannot be ruled out. Future prospective studies incorporating serial thyroid profiling, structural imaging, and comprehensive clinical stratification are heavily warranted to validate these early neuroendocrine interactions.

Comparison with previous literature further clarifies the scope of the present findings. Earlier studies have linked low T3 syndrome with mortality, heart failure, and adverse cardiovascular outcomes in acute coronary syndromes, thereby emphasizing its prognostic importance in broader clinical settings. In contrast, the present study was not designed to assess mortality, ventricular dysfunction, or long-term outcomes, but rather to explore admission-phase associations between thyroid function parameters and biochemical markers of myocardial injury. The present results therefore add to the understanding of early neuroendocrine responses in AMI, but they should not be interpreted as evidence that thyroid-function abnormalities independently predict long-term clinical prognosis in this cohort.

The study has several distinct strengths, including the consecutive enrollment of patients, early biological sampling during the acute phase (within six hours of admission), and the utilization of highly standardized chemiluminescent immunoassay methods. At the same time, important limitations must be acknowledged. These include the single-center setting, a relatively modest sample size, the absence of serial hormonal measurements to track dynamic changes over time, the lack of reverse T3 estimation, and the absence of structural imaging, echocardiographic parameters, inflammatory markers, and long-term longitudinal clinical outcomes. These limitations restrict both absolute mechanistic interpretation and widespread clinical generalizability. Furthermore, our characterization of baseline clinical parameters presents specific boundaries. While primary demographic confounders-specifically age and sex-were tracked and statistically adjusted for within our multivariable regression model, certain acute clinical confounders could not be uniformly tracked across the cohort database. These include the precise distribution of STEMI versus NSTEMI presentation, Killip class stratification, emergency reperfusion strategies, and acute pharmacotherapy exposure. These unmeasured baseline variations represent important confounding factors that can independently influence both acute thyroid-axis fluctuations and cardiac biomarker kinetics; therefore, our findings must be interpreted strictly within these clinical constraints.

## Conclusions

While our findings demonstrated a significant association between TSH and CK-MB at admission, as well as independent associations of log-transformed TSH (β = 0.33, B = 0.48, p = 0.001) and log-transformed fT3 (β = 0.28, B = 0.34, p = 0.003) with log-transformed troponin I in the multivariable linear regression model (R² = 0.385; adjusted R² = 0.352; F = 11.78; p < 0.001), these results should be interpreted with caution. Because the study was based on a cross-sectional evaluation of biochemical markers obtained at a single time point, no causal relationship can be inferred, and these findings cannot be assumed to directly reflect infarct size, ventricular dysfunction, or long-term clinical prognosis. Furthermore, direct measures of myocardial structural damage, including echocardiographic parameters, left ventricular ejection fraction, cardiac magnetic resonance imaging, inflammatory markers, and longitudinal clinical outcomes, were not evaluated. Therefore, the observed thyroid-axis alterations should be regarded as acute biochemical correlates of the initial ischemic event rather than established prognostic indicators. Accordingly, the conclusions of the present study should remain conservative: thyroid-axis alterations were independently associated with biochemical markers of myocardial injury at admission, but their clinical and prognostic significance requires confirmation in larger studies incorporating structural imaging, echocardiographic assessment, inflammatory biomarkers, and long-term clinical outcomes.

The future scope of this work includes conducting larger, multicenter studies involving more diverse patient populations to improve the generalizability and statistical robustness of these findings. Future investigations should incorporate more comprehensive clinical characterization, including STEMI versus NSTEMI status, Killip class, hemodynamic status, reperfusion strategy, emergency pharmacotherapy, and conventional cardiovascular risk factors such as hypertension, diabetes mellitus, smoking, and dyslipidemia, as these variables may influence both thyroid function and cardiac biomarker profiles. Serial assessment of thyroid hormones at admission, 24 hours, 48 hours, and discharge would help determine whether low T3 syndrome represents a transient adaptive response to acute illness or a sustained endocrine disturbance. In addition, future studies should include reverse T3 estimation, echocardiographic evaluation, and advanced cardiac imaging to clarify whether thyroid-axis abnormalities are associated with ventricular dysfunction, infarct burden, or impaired myocardial recovery. Long-term follow-up studies are also warranted to determine whether low T3 syndrome predicts heart failure, recurrent cardiovascular events, or mortality following AMI. If these associations are consistently confirmed, interventional clinical trials may be considered to evaluate whether short-term thyroid hormone replacement in carefully selected patients with low T3 syndrome can improve myocardial recovery and long-term clinical outcomes.
